# Pain management after ambulatory surgery: a prospective, multicenter, randomized, double-blinded parallel controlled trial comparing nalbuphine and tramadol

**DOI:** 10.1186/s12871-020-01125-4

**Published:** 2020-08-15

**Authors:** Yu-jiao Guan, Lai Wei, Qin Liao, Qi-wu Fang, Nong He, Chong-fang Han, Chang-hong Miao, Gang-jian Luo, Han-bing Wang, Hao Cheng, Qu-lian Guo, Zhi-gang Cheng

**Affiliations:** 1 Department of Anesthesiology, Xiangya Hospital of Central South University, No. 87 Xiangya Road, Changsha, Hunan China; 2 Department of Anesthesiology, Hunan Provincial People’s Hospital, Changsha, Hunan China; 3 Department of Anesthesiology, Third Xiangya Hospital of Central South University, Changsha, Hunan China; 4 Department of Anesthesiology, Pain Medicine & Critical Care Medicine, Aviation General Hospital of China Medical University & Beijing Institute of Translational Medicine, Chinese Academy of Sciences, Beijing, China; 5 Department of Anesthesiology, Peking University Shougang Hospital, Beijing, China; 6 Department of Anesthesiology, Shanxi Academy of Medical Sciences, Shanxi Dayi Hospital, Shanxi, China; 7 Department of Anesthesiology, Fudan University Shanghai Cancer Center, Shanghai, China; 8 Department of Anesthesiology, Third Affiliated Hospital of Sun Yat-Sen University, Guangzhou, Guangdong China; 9 Department of Anesthesiology, First People’s Hospital of Foshan, Foshan, Guangdong China; 10 Department of Anesthesiology, Beijing Ditan Hospital Capital Medical University, Beijing, China

**Keywords:** Nalbuphine, Tramadol, Ambulatory surgery, Postoperative analgesia, Anesthesia, Pain

## Abstract

**Background:**

Postoperative pain in ambulatory surgery is a multifactorial issue affecting patient satisfaction, time of discharge, and rehospitalization. This study evaluated the efficacy and safety of nalbuphine for the treatment of postoperative pain after ambulatory surgery, relative to tramadol.

**Methods:**

This multi-center, randomized, double blind, and controlled study was conducted at 10 centers. In accordance with the inclusion criteria, 492 ambulatory surgery patients were recruited. These patients had moderate to severe pain after ambulatory surgery, with a visual analogue scale (VAS) score > 3 cm. They were randomly divided into an experimental (*n* = 248) or control (*n* = 244) group and treated for analgesia with 0.2 mg/kg of nalbuphine or 2 mg/kg of tramadol, respectively. VAS scores, adverse events, and vital signs of the patients were recorded before administration (baseline; T_1_); and 30 min (T_2_), 2 h (T_3_), 4 h (T_4_), and 6 h (T_5_) after administration of analgesia. A decrease in pain intensity of more than 25% compared with the baseline was used as an indicator of analgesic efficacy. The experimental and control groups were compared with regard to this indicator of efficacy at each timepoint.

**Results:**

The VAS scores of the experimental and control groups were statistically comparable at timepoints T_1_-T_4_. At T_5_, the VAS scores of the experimental group were significantly lower than that of the control. The pain intensity was significantly higher in the experimental group compared with the control at T_2_ and T_3_. Adverse events and vital signs were similar for the two groups at each timepoint.

**Conclusions:**

Nalbuphine can provide effective and safe pain relief in patients after ambulatory surgery.

**Trial registration:**

The registration number is ChiCTR-IOR-16010032, the date of registration was 2016-11-28.

## Background

Postoperative pain is a multifactorial issue that may result in patient dissatisfaction, delayed discharge, and unanticipated hospital admission after ambulatory surgery [1]. Both delayed discharge and unanticipated hospital admission have the undesirable effect of increasing healthcare costs [2]. In the postoperative period, moderate to severe pain are frequently observed during the first 24 to 48 h after ambulatory surgery [3].

Patient recovery after ambulatory surgery has improved since the introduction of the concept of enhanced recovery after surgery, a multimodal perioperative care pathway designed to achieve early recovery after surgery [4]. Ambulatory surgery has significantly shortened hospitalization, accelerated turnover, and reduced hospital costs and rates of nosocomial infections [5]. However, the shortened hospitalization and increased mobility of surgical patients have necessitated the need to improve the efficacy of anesthesia and perioperative management. Therefore, postoperative pain and the complications arising from its treatment are important considerations for patients undergoing ambulatory surgery.

Various drugs have been used to prolong postoperative analgesia, such as tramadol [6], ketorolac [7], dexmedetomidine [8], ketamine [9], and nalbuphine [10]. Nalbuphine, a synthetic opioid agonist-antagonist analgesic, is primarily a kappa (κ) agonist and a partial mu (μ) antagonist. It has a better safety profile with fewer side effects compared with other opioids, because of its agonist and antagonist activities [11]. Nalbuphine [12] exerts its analgesic and hypnotic effects through its κ opioid receptor, which may reduce μ opioid receptor-related adverse events. Numerous studies [13, 14] have reported its advantages in pain management.

There have been few studies in China of nalbuphine for the treatment of postoperative pain after ambulatory surgery. The present study evaluated the analgesic efficacy and safety of intravenous nalbuphine hydrochloride, relative to tramadol, for the treatment of postoperative pain after ambulatory surgery, including a non-inferiority control trial.

## Methods

### Participants

This study was approved by the Ethics Committee of Xiangya Hospital of Central South University (IRB 201608066). Written informed consent was obtained from all subjects participating in the trial. The trial was registered prior to patient enrollment at chictr.org.cn (ChiCTR-IOR-16010032, Principal investigator: Qulian Guo, Date of registration: 2016-11-26).

A multicenter, prospective, randomized, parallel-controlled, double-blinded study for pain management after ambulatory surgery in adult patients was undertaken in 10 hospitals. Patients were screened at each center. The study was reported in accordance with the guidelines of the Consolidated Standards of Reporting Trials (CONSORT).

The patient inclusion criteria were as follows: 18 to 65 years old; ASA (American Society of Anesthesiologists) I-II; with postoperative pain after surgeries of the breast (except radical surgery for mastocarcinoma) or thyroid, or hysteroscopy, or laparoscopic cholecystectomy; operative time < 2 h; visual analog scale (VAS) score < 3 cm before the surgery, and VAS score > 3 cm after recovery from anesthesia; body mass index (BMI) 18–29 kg/m^2^, and signed informed consent.

Patients were excluded from this study if they were allergic to the medication or any of the excipients in the product. Patients with current or histories of any of the following were also excluded: opioid allergy; acute or chronic alcoholism or drug addiction; neurological disease; opioid used within the last 3 months; paralytic ileus; increased intracranial pressure or head injury; chronic opioid use (taking opioids for more than 3 months); hypotension; hypothyroidism, asthma (to be avoided during seizure); hypertrophy of the prostate; epilepsy; coronary heart disease; bronchial asthma; respiratory insufficiency; or respiratory failure. Patients taking or who had taken monoamine oxidase inhibitor or antidepressants within the past 15 days were excluded. Patients with abnormal preoperative liver and kidney function were also excluded, defined as abnormal alanine aminotransferase (ALT), aspartic aminotransferase (AST), blood urea nitrogen (BUN) or creatinine (Cr) (ALT and AST > 1.5 times the normal limit, and BUN and Cr higher than the normal limit); coronary heart disease; bronchial asthma; respiratory insufficiency or respiratory failure; or poorly controlled or difficult hypertension. The latter was defined as systolic blood pressure (SBP) ≥ 160 mmHg or diastolic pressure (DBP) ≥ 100 mmHg. In addition, patients with any of the following were excluded: pregnancy; abnormal coagulation function; participation in another medication trial within the previous 30 days; unable to express their intention correctly; poor compliance; unable to complete the study program; or anyone the researchers considered inappropriate to participate.

### Trial design

Patients were randomly assigned to receive either the experimental (group E) or control (group C) treatment in the postoperative period. Group E was treated with nalbuphine hydrochloride (1,161,101 Yichang Humanwell Pharmaceutical) diluted with saline to 1 mg/L. Group C was administered tramadol hydrochloride diluted with saline to 10 mg/L.

The study medication was selected and prepared according to a random number list (nalbuphine hydrochloride or tramadol hydrochloride). The study was blinded, by excluding the researcher who prepared the postoperative medications from participating in test observations and follow-ups. The researchers involved in observation and evaluation of the experiment, and patients and doctors, were blinded throughout the study.

### Interventions

All patients were administered intravenously with 5 mg of dexamethasone before induction of general anesthesia, and 8 mg of ondansetron at the time of surgery completion, to prevent postoperative nausea and vomiting. The bispectral index (BIS) value was maintained between 40 and 60 during the operation. Anesthesia induction was performed using sufentanil (0.5 μg/kg) and propofol (2–2.5 mg/kg), with cisatracurium (0.1–0.2 mg/kg) given when necessary. Anesthesia was maintained by simultaneous infusion of propofol and remifentanil (0.1–0.15 μg/kg/min). An additional 0.1 mg/kg of cisatracurium was added intraoperatively when required. Intraoperative fluid infusion and other anesthetic management were performed routinely.

After the surgery, patients who were fully awake and feeling pain for the first time were assessed for pain while at rest, using the VAS. If the VAS score was > 3 cm, the patients were included in the study and the pain score was used as the baseline (T_1_). The test medications (nalbuphine hydrochloride or tramadol hydrochloride) were administered at 0.2 mL/kg. The VAS at rest was used to evaluate the efficacy of the medications and was recorded before administration (T_1_), and after administration at 30 min (T_2_), 2 h (T_3_), 4 h (T_4_), and 6 h (T_5_). The following vital signs were recorded at each timepoint: SBP, DBP, mean arterial pressure (MAP), heart rate, and respiratory rate. Adverse events and any medications used were also recorded.

Within 2 h after administration of the medications, if the VAS score was > 3 cm, it was deemed that the analgesic effect was invalid, and the patient was discontinued from the trial. One hundred milligrams of flurbiprofen axetil was infused intravenously as a rescue analgesia, and the name and dose were recorded. The use of other analgesics aside from those involved in the study, such as opioids, tranquilizers, anesthetics and antiemetics, were prohibited during the study period. If other analgesics were required to control the pain, the patient was discontinued.

### Outcomes

#### Primary outcome

The pain intensity was measured using the VAS. A decrease in VAS score of more than 25% compared with the baseline was used as an indicator of analgesic efficacy [15]. The VAS score was also compared between groups E and C at all timepoints to determine any differences in the efficacy and duration of the analgesic effects.

#### Secondary outcome

The vital signs (SBP, DBP, respiratory rate and heart rate) were measured and used as safety indicators. The vital signs were also compared between groups E and C, and within each group, at each timepoint. Any differences observed could be used as a secondary indicator to determine analgesic efficacy.

#### Adverse events

Adverse events such as medication extravasation, dizziness, nausea, vomiting, and hidrosis were recorded during the study. The rates of adverse events was compared between groups E and C to determine the effects of the treatments.

#### Sample size

Sample size was calculated by VAS at rest at each timepoint. Based on a previous report [16], a single intravenous injection of tramadol was administered to patients with postoperative pain after day surgery, and the VAS score was ~ 2.43 cm at 30 min after administration. Assuming that the analgesic effect of nalbuphine was better than tramadol, with α = 0.05 and β = 0.2, the VAS score difference between the two groups (μ_A_ − μ_B_) would be 0.5 and the standard deviation σ = 1.7. The sample size(n) was calculated using the formula [17]:

$$ n=2{\left[\alpha \left({z}_{1-\raisebox{1ex}{$\alpha $}\!\left/ \!\raisebox{-1ex}{$2$}\right.}+{z}_{1-\beta}\right)/\left({\mu}_{\mathrm{A}}-{\mu}_{\mathrm{B}}\right)\right]}^2 $$

Each group required 182 subjects and with consideration of the estimated dropout rate, 250 patients were included in each group. Therefore, 500 patients were recruited in this study, with 50 patients in each center.

#### Statistical methods

Descriptive statistics were used to describe all demographic data. The *t*-test was applied to analyze the changes in VAS scores between the two treatment groups at each timepoint, and at different timepoints relative to the baseline. The Wilcoxon test was used to analyze the pain classification of patients at each observation timepoint. The pain intensity between the two groups was compared using the chi-squared (χ^2^) test. *P* < 0.05 was considered statistically significant. The incidence of adverse events, changes in blood pressure, respiratory rate, and heart rate relative to the baseline at each timepoint, and differences between the groups, were analyzed using the *t*-test.

## Results

### Participants

**Fig. 1:**
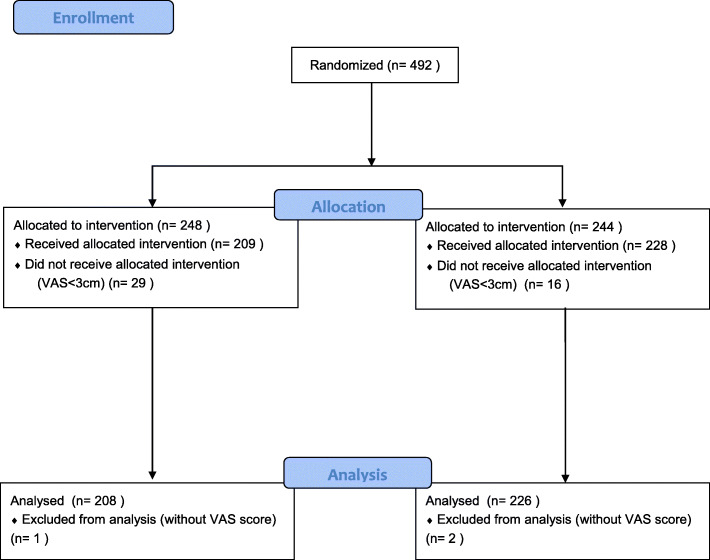
CONSORT flow diagram of progress through the phases of a randomized, double-blinded parallel controlled trial of the 2 groups

The study population comprised 492 randomly coded patients recruited from 10 centers (Fig. 1). However, 55 patients were excluded as they did not meet the eligibility criteria of a VAS score < 4 cm. Thus, the trial consisted of 437 patients: 209 in group E, and 228 in group C.

### Baseline data

The differences in age and gender between groups E and C were not statistically significant (Table 1). The results of the preoperative test, physical examination, and medical histories of the two groups were relatively similar, with no statistical difference. There were no statistically significant differences in the types of surgery between the two groups (Table 2). There were also no differences in the use of opioids including sufentanil and remifentanil between the two groups, during surgery.

**Table 1:** Patient demographics of groups E and C^a^

	Group E	Group C	*P*
Subjects, n	209	228	
Gender, n (%)			
Male	52 (25.0)	48 (21.1)	0.340
Female	156 (75.0)	179 (78.9)	
Age, y	40.66 ± 12.04	40.67 ± 11.81	0.994
BMI, kg/m^2^	23.15 ± 2.84	23.20 ± 2.87	0.847
Respiration, rpm	17.09 ± 1.81	17.04 ± 2.18	0.808
Heart rate, bpm	74.45 ± 8.43	74.48 ± 9.75	0.978
Heart rhythm, n (%)			
Normal	207 (99.0)	225 (99.1)	1.000
Abnormal	2 (1.0)	2 (0.9)	
SBP, mmHg	121.63 ± 16.26	122.64 ± 16.01	0.513
DBP, mmHg	74.92 ± 9.48	74.63 ± 8.80	0.738
MAP, mmHg	92.91 ± 11.65	93.17 ± 10.84	0.806

^a^Group E was treated with nalbuphine hydrochloride diluted with saline to 1 mg/L. Group C was administered tramadol hydrochloride diluted with saline to 10 mg/L.

**Table 2:** Types of surgery, n (%)^a^

	Total, n	Breast	Thyroid	Hysteroscopy	LC	Others
Group E	209	27 (12.9)	41 (19.6)	39 (18.7)	91 (43.5)	11 (5.3)
Group C	228	26 (11.4)	45 (19.7)	44 (19.3)	106 (46.5)	7 (3.1)

^a^Reported as n (%), unless indicated otherwise. Group E was treated with nalbuphine hydrochloride diluted with saline to 1 mg/L. Group C was administered tramadol hydrochloride diluted with saline to 10 mg/L. Other surgeries included: lumbar disc exploration, laparoscopic gastric perforation repair, endoscopic sinus surgery, surgical removal of internal fixation of fractured bones.LC, laparoscopic cholecystectomy

During the observation period, 14 (6.3%) and 20 (9.0%) patients in groups E and C, respectively, were treated with rescue analgesic medication consisting of 100 mg of flurbiprofen axetil. There was no statistically significant difference between the two groups with regard to the percentage using rescue analgesic medication (χ^2^ = 1.206; *P* = 0.272). There was no significant deviation from the regimen for all concomitant and combination medications and no statistically significant difference between the two groups.

### Outcomes

#### Primary outcome

**Fig. 2:**
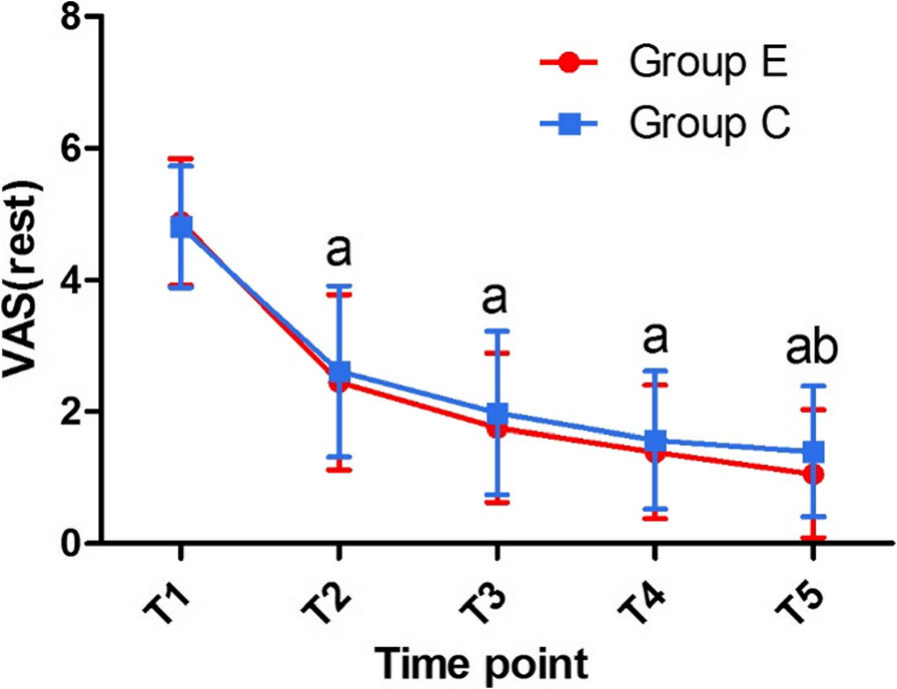
The VAS at rest in the experimental (Group E) and control group (Group C). Time points: T_1_: before administration; T_2_: after administration at 30 min; T_3_: after administration at 2 h; T_4_: after administration at 4 h; T_5_: after administration at 6 h. ^a^After administration, the VAS of Group E was lower than that of Group C, from T_2_-T_5_; ^b^there was a statistically significant difference in VAS between the 2 groups at T_5_. Data are expressed as mean ± standard deviation

A pairwise comparison of the VASs determined at rest at different timepoints between groups E and C revealed no difference between the VAS scores at T_1_, T_2_, T_3_, or T_4_, respectively. However, at T_5_ the VAS at rest of group E was significantly lower than that of group C (Fig. 2). A decrease in pain intensity of more than 25% compared with the baseline (T_1_) was used as an indicator of analgesic efficacy (Table 3). The analgesic efficacy experienced by group E at T_2_ and T_3_ was significantly higher than that of group C.

**Table 3:** Pain reduction when compared to baseline (T_1_), *n* (%)^a^

		Group E^b^	Group C^c^	χ^2^	*P*
T_2_^d^	Effective	186 (89.0)	178 (78.4)	8.837	0.003
	Noneffective	23 (11.0)	49 (21.6)		
T_3_^e^	Effective	192 (97.0)	195 (90.7)	6.874	0.009
	Noneffective	6 (3.0%)	20 (9.3%)		
T_4_^f^	Effective	190 (97.4)	203 (97.1)	0.036	0.850
	Noneffective	5 (2.6)	6 (2.9)		
T_5_^g^	Effective	189 (97.9)	201 (97.6)	0.000	1.000
	Noneffective	4 (2.1)	5 (2.4)		

^a^Effective pain reduction is defined as a decrease in pain intensity > 25%, compared with the baseline (T_1_). Noneffective is defined as a decrease in pain intensity < 25%, compared with the baseline (T_1_)

^b^Group E (n = 209) was treated with nalbuphine hydrochloride diluted with saline to 1 mg/L.

^c^Group C (n = 228) was administered tramadol hydrochloride diluted with saline to 10 mg/L.

^d^T_2_: after administration at 30 min

^e^T_3_: after administration at 2 h

^f^T_4_: after administration at 4 h

^g^T_5_: after administration at 6 h

#### Adverse events

Adverse events occurred in 6 (2.9%) subjects in group E and 3 (1.3%) subjects in group C, with no serious adverse events or deaths occurring in either group. The number of adverse events was higher in group E compared with group C, but the difference was not statistically significant (Table 4).

**Table 4:** Patients experiencing adverse events, *n*

	Group E	Group C
Total subjects experiencing adverse events	6	3
Vasculitis, medication extravasation	1	0
Dizziness, nausea, vomiting	5	2
Hidrosis	0	1

#### Secondary outcome

The vital signs (SBP, DBP, respiratory rate, and heart rate) of groups E and C at all timepoints were statistically similar (Table 5). For both groups, the mean SBP, DBP, and heart rate at each of the timepoints T_2_, T_3_, T_4_, and T_5_ were significantly lower than at T_1_. However, the blood pressures at T_2_ to T_5_ were comparable to that at admission (T_0_), and there was no significant difference in respiratory rates.

**Table 5:** Vital signs at each timepoint ^a^

	Vital signs	Group E^b^	Group C^c^	*P*
T_0_	SBP	121.63 ± 16.26	122.64 ± 16.01	0.513
	DBP	74.92 ± 9.48	74.63 ± 8.80	0.738
	Respiratory rate	17.09 ± 1.81	17.04 ± 2.18	0.808
	Heart rate	74.45 ± 8.43	74.48 ± 9.75	0.978
T_1_	SBP	128.96 ± 17.74**	130.18 ± 17.05**	0.465
	DBP	79.04 ± 11.32**	78.85 ± 11.45**	0.864
	Respiratory rate	16.52 ± 2.40	16.89 ± 2.56	0.121
	Heart rate	78.69 ± 16.33	78.89 ± 14.97	0.893
T_2_	SBP	123.87 ± 16.07*	126.10 ± 16.68*	0.157
	DBP	76.20 ± 9.88*	77.12 ± 12.16*	0.390
	Respiratory rate	16.74 ± 2.19	17.12 ± 2.46	0.092
	Heart rate	76.82 ± 13.95*	76.54 ± 12.06*	0.822
T_3_	SBP	118.37 ± 15.23*	121.18 ± 15.53*	0.065
	DBP	72.82 ± 9.19*	74.00 ± 9.70*	0.209
	Respiratory rate	16.53 ± 1.85	16.72 ± 2.13	0.321
	Heart rate	74.06 ± 10.82*	74.59 ± 9.58*	0.598
T_4_	SBP	116.72 ± 15.43*	118.82 ± 15.19*	0.169
	DBP	71.27 ± 9.36*	72.07 ± 9.73*	0.399
	Respiratory rate	16.44 ± 1.78	16.62 ± 2.20	0.366
	Heart rate	72.81 ± 9.44*	73.62 ± 8.99*	0.378
T_5_	SBP	116.53 ± 14.86*	117.11 ± 14.46*	0.691
	DBP	70.64 ± 9.39*	70.63 ± 9.45*	0.986
	Respiratory rate	16.46 ± 1.84	16.54 ± 2.07	0.682
	Heart rate	72.65 ± 9.35*	72.76 ± 9.22*	0.905

^a^T_0_: at admission, T_1_: before administration, T_2_: after administration at 30 min, T_3_: after administration at 2 h, T_4_: after administration at 4 h, T_5_: after administration at 6 h

^b^Group E (*n* = 209) was treated with nalbuphine hydrochloride diluted with saline to 1 mg/L.

^c^Group C (*n* = 228) was administered tramadol hydrochloride diluted with saline to 10 mg/L.

*Difference is statistically significant compared with T_1_; **difference is statistically significant compared with T_0_

## Discussion

In this prospective, multicenter study, 437 patients were randomized to receive either nalbuphine (group E) or tramadol (group C) to treat pain after ambulatory surgery. Group E experienced significantly longer duration of analgesia compared with group C. At each timepoint, the vital signs (SBP, DBP, respiratory rate, and heart rate) of the 2 groups were statistically comparable. However, within each group there were significant differences in SBP, DBP, and heart rate at T_2_, T_3_, T_4_, and T_5_, relative to T_1_. Overall, the analgesic effect of nalbuphine was comparable to that of tramadol, with nalbuphine having a longer duration of analgesia.

In China, the number of day surgeries is increasing due to improvements in surgery and anesthesia, with shorter recovery time and patients discharged within 24 h after surgery. Therefore, there is a higher demand for anesthesia and a need to improve the quality of analgesics. While achieving rapid recovery, patients also need to avoid complications related to surgery and anesthesia, such as pain, nausea, and vomiting. Numerous studies [17, 18] have shown that after day surgery nearly 80% of patients experience pain. Postoperative pain not only affects patients’ rehabilitation and prolongs hospitalization, it can also result in progression from acute to chronic pain, which is the main cause of readmission after day surgery [19].

According to the Chinese Society of Anesthesiology [20], systemic opioids given to patients undergoing ambulatory surgery with general anesthesia activate opioid receptors and stimulate various organs. This often results in nausea and vomiting, pruritus, urinary retention, excessive sedation and respiratory inhibition. Thus, in principle, systemic opioids are not used for postoperative pain relief after day surgery. The analgesic and adverse reactions of mixed agonist-antagonist opioids, such as nalbuphine and dezocine, also exhibit a ceiling effect. Implementation of multimodal analgesia using NSAIDs can significantly reduce the dose of opioid and adverse reactions, and can be used postoperatively to manage moderate pain after ambulatory surgery.

Nalbuphine, a mixed agonist-antagonist opioid, is associated with milder μ receptor-related side effects. Its plasma half-life is 5 h, and in clinical studies the duration of analgesic activity ranges from 3 to 6 h [21]. In our study, the VAS at rest of group E was less than 4 points, and the difference was statistically significant compared with the VAS at rest before administration. This indicates that nalbuphine could effectively relieve pain after ambulatory surgery. Similar results were also observed in animal studies that showed amelioration of somatic and visceral pain in mice after treatment with nalbuphine [22].

In the present study, the VAS at rest at timepoints T_1_ to T_4_ of the nalbuphine group (group E) did not differ from that of the control. At T_5_, the VAS at rest of the nalbuphine group was significantly lower than that of the tramadol group. This indicates that the duration of nalbuphine for pain relief after ambulatory surgery was longer than that of tramadol. There were 6 cases (2.8%) of adverse reactions in the nalbuphine group, which was not significantly different from the 3 cases (1.3%) in the tramadol group.

The incidence of adverse reactions associated with nalbuphine is relatively low compared with other opioid medications. A meta-analysis of randomized controlled trials by Zeng et al. [23] showed that nalbuphine has similar analgesic effects compared to morphine, and a better drug safety profile with a low incidence of postoperative pruritus, respiratory inhibition, nausea, and vomiting. In addition, studies have reported that antagonism of the μ receptor by nalbuphine could reduce the adverse reactions of other opioids, as seen in the combination of morphine and nalbuphine in patient-controlled analgesia or patient-controlled epidural analgesia [24, 25]; and the rate of adverse effects such as urinary retention related to morphine, pruritus, and nausea was significantly less. Nalbuphine with sufentanil used in patient-controlled analgesia could reduce the incidence of opioid-related nausea and vomiting and improved patients’ satisfaction with analgesia [26, 27].

In the present study, the difference in respiratory rates before and after administration in both the nalbuphine and tramadol groups was not statistically significant, and no respiratory depression was observed. Many studies have reported that respiratory depression caused by nalbuphine is small and has a ceiling effect [28, 29]. In one study, a neonate was wrongly administered a ten-fold higher dose than required of nalbuphine, and it resulted in only prolonged sedation with no respiratory failure [30].

Studies have shown that pre-anesthetic injections of nalbuphine could reduce stress responses and fluctuations in blood pressure and heart rate during intubation [31, 32]. In the present study, the blood pressures and heart rates of both groups after administration were significantly lower than at T_1_, although still within normal ranges. The blood pressure at T_0_ (Inception of the study) was compared to the blood pressure after surgery (T_1_-T_5_); the blood pressure at T_1_ was significantly higher than at T_0_. However, at the later timepoints, T_2_ to T_5_, there were no statistical differences in the blood pressures compared to T_0_. The decrease in blood pressure after administration (T_2_-T_5_) may have been due to the alleviation of pain. If so, then the lowered blood pressure could also indicate the analgesic efficacy of nalbuphine.

There are several limitations in this study. First, a limited number of parameters (VAS score, adverse events, and change of vital signs) were observed within the half-life of the medication. Secondly, the VAS scores were recorded at rest and not during movement. Finally, due to ethical issues a placebo control group was not possible. Therefore, we were not able to assess the effectiveness of nalbuphine or tramadol at 4 and 6 h after administration. Fortunately, none of the patients dropped out during the 4 or 6 h after administration of medication for pain. However, the present results warrant further experiments to determine comprehensively the effectiveness and safety of nalbuphine for the treatment of pain after ambulatory surgery.

## Conclusion

This study indicates that nalbuphine at a recommended dose of 0.2 mg/kg is safe and effective for pain management after ambulatory surgery.

## Data Availability

The datasets generated and analyzed during the present study are available from the corresponding author on reasonable request.

## Abbreviations

ALT: alanine aminotransferase
ASA: American Society of Anesthesiologists
AST: Aspartic aminotransferase
BIS: Bispectral index
BMI: Body mass index
BUN: Blood urea nitrogen
C: Control
CONSORT: Consolidated Standards of Reporting Trials
Cr: Creatinine
DBP: Diastolic pressure
E: Experimental
MAP: Mean arterial pressure
SBP: Systolic blood pressure
VAS: Visual analog scale
